# Genetic Analysis of Days Open in Moroccan Holstein Using Different Models to Account for Censored Data

**DOI:** 10.3390/ani14243614

**Published:** 2024-12-15

**Authors:** Narjice Chafai, Bouabid Badaoui

**Affiliations:** 1 Laboratory of Biodiversity, Ecology and Genome, Department of Biology, Faculty of Sciences, Mohammed V University in Rabat, B.P. 1014 RP, Rabat 10100, Morocco; 2 African Sustainable Agriculture Research Institute (ASARI), Mohammed VI Polytechnic University (UM6P), Laayoune 70000, Morocco; bouabidbadaoui@gmail.com

**Keywords:** fertility, days open, censored records, genetic parameters, threshold linear model, penalty methods

## Abstract

**Simple Summary:**

Reproductive performance is a critical factor for the economic success and long-term viability of dairy herds. Intense selection for milk production has resulted in a decline in fertility traits in Holstein cows, leading to negative consequences for the industry. To combat this, incorporating fertility traits into the genetic evaluation is a potential solution. However, fertility data are often incomplete due to a variety of reasons; in this study, which has a limited dataset, it is essential to address this issue. The study utilized three methods to handle censorship: a linear model, a penalty method, and a threshold linear model with a penalty. The findings revealed that the penalized threshold model showed a slightly higher heritability compared to linear models. Moreover, both the penalty method and the threshold method exhibited comparable predictive abilities and substantial overlap in common animals, suggesting that both methods can be employed to impute days open censored data in this population.

**Abstract:**

Reproductive efficiency is a key element of profitability in dairy herds. However, the genetic evaluation of fertility traits is often challenged by the presence of high censorship rates due to various reasons. An easy approach to address this challenge is to remove the censored data from the dataset. However, removing data might bias the genetic evaluation. Therefore, addressing this issue is crucial, particularly for small populations and populations with limited size. This study uses a Moroccan Holstein dataset to compare two Gaussian linear models and a threshold linear model to handle censored records of days open (DO). Data contained 8646 records of days open across the first three parities of 6337 Holstein cows. The pedigree file comprised 11,555 animals and 14.51% of the dataset was censored. The genetic parameters and breeding values of DO were computed using three different methods: a linear model where all censored records were omitted (LM), a penalty method in which a constant equal to one estrus cycle in cattle was added to the maximum value of DO in each contemporary group to impute the censored records (PLM), and a bivariate threshold model with a penalty (PTM). The heritability estimates were equal to 0.021 ± 0.01 (PLM), 0.029 ± 0.01 (LM), and 0.033 ± 0.01 (PTM). The penalty method and the threshold linear model with a penalty showed better prediction accuracy calculated using the LR method (0.21, and 0.20, respectively). PLM and PTM had a high Spearman correlation (0.99) between the estimated breeding values of the validation dataset, which explains the high percentage of common animals in the top 20% of selected animals. The lack of changes in the ranking of animals between PLM and PTM suggests that both methods can be used to address censored data in this population.

## 1. Introduction

Fertility traits are critical in the genetic evaluation of dairy cattle as they directly influence reproductive efficiency, which is essential for the sustainability and profitability of dairy operations. Effective female fertility is defined as the ability to display estrus and achieve pregnancy with minimal inseminations [1]. A reduced number of inseminations translates to lower veterinary and hormonal treatment costs, reduced semen expenses, and diminished labor associated with artificial insemination. Due to intensive selection for milk production, particularly in the Holstein breed, and the negative correlation between milk yield and fertility traits, the reproductive performance of these cows has significantly declined [2]. Implementing genetic evaluations incorporating milk and fertility traits enables breeders to select animals with superior productive performance without greatly compromising reproduction [3]. In the past decades, most breeding companies shifted their emphasis from milk production to including functional traits through selection indices. This approach could potentially improve herd fertility, enhance genetic progress, and yield greater economic returns. For instance, Scandinavian countries included health, fertility, and longevity traits in their selection indices for over two decades. Consequently, the decline in mean performance for such traits was stabilized [4]. Similarly, in 2003, the US introduced genetic evaluations for daughter pregnancy rates to improve fertility in dairy cows. This evaluation used DO data and transformed it into 21-day pregnancy rates as a measure of reproductive efficiency. As a result, significant improvements were observed with notable differences in fertility between the sires [4].

Fertility trait field data contain high noise levels due to several factors [5], including preferential treatments for different individuals and varying management practices. For example, avoiding breeding cows during hot seasons, when the success of insemination is very low due to heat stress, can introduce variability. Additionally, missed heat detections unfairly penalize cows for not conceiving, further contributing to the noise in fertility data. On top of that, fertility data generally comprise censored records as a result of retrieving the datasets before some cows conceive or give birth. Consequently, there are generally a lot of missing records due to censoring for days open (DO), defined as the days from calving to the following conception. This issue can be addressed by simply removing all the censored records. This approach offers a straightforward solution to this issue and computation simplicity, but it may also compromise the efficiency and accuracy of the estimation process and bias the genetic evaluation [6]. An alternative approach to tackle this problem is by imputing these censored records. Several methods have been proposed to address this challenge. The penalized Gaussian linear model (PLM) is one approach that assigns a penalty to each censored record. In this method, the highest value within each contemporary group is added to censored records [7]. The constant value of 21 days is included to account for the duration of an estrus cycle in cattle. This approach assumes that any female who failed to conceive would have conceived if given an extra cycle [8]. The penalty method has been considered suitable to treat censored records in the genetic evaluations of fertility-related traits [9]. Data augmentation techniques can also be utilized for censored data imputation. One such model is the linear-threshold approach [7]. This method proposes a mixed effects model that incorporates a latent variable, liability (l), for each observation. If a record is censored and the pregnancy status of the cow with the censored record was non-pregnant at the last insemination, then its corresponding liability must be greater than a specified threshold T. The penalized threshold model is similar to the linear threshold model but assigns a penalty for censored records. As previously discussed, [6], the highest value in each contemporary group was identified, and a constant of 21 days was added to each censored record. Additionally, a latent variable indicating the censorship status was incorporated. The Bayesian alternative provides an appealing solution for censored data. However, the complex models and data structures typically found in animal breeding records can present computational and numerical challenges that are not yet fully understood [10]. Overall, the potential advantage of threshold-linear models over linear models with censoring in assessing fertility traits has not been quantified [11].

Datasets on reproductive traits in Moroccan Holstein suffer from high rates of censorship. Furthermore, the size of fertility trait datasets is generally limited. To perform genetic evaluations to choose elite dams’ progeny for replacement, imputing censored records is crucial. The primary objective of this study is to apply various censoring models and evaluate their predictive ability to potentially identify the best model to address the issue of censored data of the DO trait within a dataset of Holstein cows in Morocco.

## 2. Materials and Methods

### 2.1. Data

Data were provided by the ‘Les Domaines Agricoles’ company. The dataset comprises records of Holstein cows raised in four herds located in two regions of northern Morocco. These regions are characterized by a Mediterranean climate, with mild winters and long, hot, and dry summers. The animals were managed under an intensive production system, where they were fed a total mixed ration and milked twice a day. Artificial insemination with frozen imported semen was the only method used by specialized technicians. Pregnant cows were kept in free stalls, which were cleaned twice per day. The farm employees kept a constant watch on the pregnant cows and groups in maternity pens, monitoring for any visual signs of parturition. Once parturition occurred, the calves were promptly removed from the maternity pens and relocated to a heated calf pen. Records of milk yield, calving dates, artificial insemination dates, conception dates, dry-off dates, and heat detection dates were recorded using herd management software.

The raw dataset included 13,501 observations collected from 2017 to 2023. These observations were distributed across four herds: Herd 1 consisted of 3812 observations from 2149 cows, Herd 2 comprised 3170 records from 2303 cows, Herd 3 included 4092 records from 2098 cows, and Herd 4 encompassed 2427 records from 1464 cows. The mean age at each calving in the raw data was 781 days for the first calving, 1231 days for the second calving, and 1666 days for the third calving. The dataset included parities up to the ninth parity. The number of observations decreased at the fourth parity to 855 and at the ninth parity to 9 observations.

To address non-random missingness, only the first three lactations were retained. Data processing involved removing cows with incorrect identification, cows that calved for the first time before 530 days of age, those with identical calving and abortion dates, and those with missing calving dates. For the linear covariates age at calving and days in milk at first insemination, records exceeding the mean plus three standard deviations were excluded. The trait of interest, days open (DO), was defined as the number of days from calving to subsequent conception. Observations of DO were retained only if they fell within the range of the mean plus or minus three standard deviations within parity. Additionally, records of DO smaller than 20 days were deleted. Contemporary groups were created by combining herd, year of calving, and season of calving (HYS). The seasons were defined as winter (December to February), spring (March to May), summer (June to August), and fall (September to November). Contemporary groups with fewer than three observations were removed. The maximum number of observations in a contemporary group was 248. Due to the size of the dataset, 287 sire had one progeny. Therefore, no restriction was applied to the number of daughters per sires. Records were assumed censored if no pregnancy was confirmed by a subsequent conception or calving.

After data processing, the dataset consisted of 8646 records from 6337 cows and 14.51% of the DO records were censored. The pedigree comprised 11,555 animals and 931 sires. The dataset with no censored records consisted of 7550 observations of 5468 cows and the pedigree file consisted of 10,375 animals.

### 2.2. Statistical Models

Two Gaussian linear models were used in this analysis, a classical linear model with no censored data and a linear model with penalty. In the first linear model (LM), the censored data were completely removed from the dataset. In matrix notation, the animal model can be expressed as

(1) y=Xβ+Zu+Wpe+e

where ***y*** is the vector of observations of DO; ***β*** is the vector of fixed effects, including the contemporary groups herd-year-season with 122 classes (HYS), parity (3 classes), and 2 linear covariates: days in milk at first insemination and age at calving; ***u*** is the vector of additive genetic effects; ***pe*** is the vector of permanent environment effect; and ***e*** is the vector of residuals. X, Z and W are known incidence matrices. The random genetic effect **u** was assumed to be normally distributed $u\sim N\left(0,\;A\sigma_{u}^{2}\right)$ where ***A*** is the numerator relationship matrix and $\sigma_{u}^{2}$ is the additive genetic variance. The permanent environment effect is also assumed to follow a normal distribution $pe\sim N\left(0,\;I\sigma_{pe}^{2}\right)$ where ***I*** is an identity matrix and $\sigma_{pe}^{2}$ is the permanent environment variance. The residuals are assumed to be independent and follow a normal distribution $e\sim N\left(0,I\sigma_{e}^{2}\right)$ where $\sigma_{e}^{2}$ is the residual variance and ***I*** is an identity matrix.

The penalty method (PLM) proposed by Johnston and Bunter [8] assigns a penalty for each censored record. Specifically, it adds the highest value of DO within each contemporary group, which is equivalent to the length of the estrous cycle in cattle at 21 days, to each censored record [7,12]. This method assumes that if these animals were given the opportunity for an additional estrous cycle, they would likely conceive. The analysis for this approach utilized the previously described animal model (1).

The threshold linear model with a penalty (PTM) is a Bayesian bivariate model in the scale of a latent variable, liability (***l***). In this approach, data were augmented using an additional binary trait corresponding to censorship status. The binary trait was equal to 1 if the record was censored and 0 otherwise. The underlying continuous liability was associated with the binary trait by the following formula:

(2) $$y_{binary,i}=\left\{\begin{array}{c} 1\;\mathrm{if}\;l_{i}>T=0\\ 0\;\mathrm{otherwise} \end{array}\right.$$

where $y_{binary,i}$ is the binary response for animal i, and the threshold T was assumed equal to 0. The liability values were updated at each iteration of the Gibbs sampler.

The threshold-linear animal model on a liability scale is given by

(3) $$\left[\begin{array}{c} y_{DO}\\ l \end{array}\right]=\left[\begin{array}{cc} X_{DO} & 0\\ 0 & X_{l} \end{array}\right]\left[\begin{array}{c} \beta_{DO}\\ \beta_{l} \end{array}\right]+\left[\begin{array}{cc} Z_{DO} & 0\\ 0 & Z_{l} \end{array}\right]\left[\begin{array}{c} u_{DO}\\ u_{l} \end{array}\right]+\left[\begin{array}{cc} W_{DO} & 0\\ 0 & W_{l} \end{array}\right]\left[\begin{array}{c} {pe}_{DO}\\ {pe}_{l} \end{array}\right]+\left[\begin{array}{c} e_{DO}\\ e_{l} \end{array}\right]$$

where $y_{DO}=[y_{o},y_{c}]$ consists of the vector of observed records of DO ($y_{o}$) and the vector of augmented records with a penalty ($y_{c}$), calculated as the maximum value of DO within each contemporary group (HYS) plus a constant equal to 21 days, as described by Costa et al. [13]; ***l*** is the censoring liability; ***β*** is the vector of fixed effects including contemporary groups, parity, age at calving, and days in milk at first insemination; ***u*** is the vector of additive genetic effect; ***pe*** is the vector of permanent environment effects; and ***e*** is the vector of residuals. $X_{DO},\;Z_{DO},\;W_{DO},\;X_{l},\;Z_{l}$, and $W_{l}$ are the incidence matrices related to the fixed effects, the additive genetic effects, and the permanent environment effects, for both DO records (observed and augmented with a penalty) and the correlated binary variable corresponding to censorship status, respectively.

For all the models, a Gibbs sampler was run for a single chain of 500,000 iterations with a burn-in of 100,000 iterations and a thinning interval of 50. The genetic parameters and breeding values were inferred using BLUPF90+ programs [14]. A burn-in period was determined using visual inspection and the thinning interval was chosen using the autocorrelations computed by the Postgibbsf90 program [14]. Convergence was assessed using the Geweke diagnostic [15] and visual inspection of trace plots.

### 2.3. Accuracy of Genetic Predictions Using the Three Models

The prediction accuracy for the three models LM, PLM, and PTM was evaluated using the LR method proposed by Legarra and Reverter [16]. The validation cohort comprised animals with phenotypes born in 2020 or later. As a result, the validation set consisted of 30% of the younger animals in the complete dataset. The breeding values were estimated using the whole dataset (${\hat{u}}_{w}$) and a reduced dataset from which the phenotypes of the younger animals (the validation set) were removed (${\hat{u}}_{p}$). The accuracies of the estimated breeding values using the LR method ${\hat{ACC}}_{LR}$ can be obtained using the following formula:

(4) $${\hat{ACC}}_{LR}=\sqrt{\frac{cov({\hat{u}}_{w},{\hat{u}}_{p})\;}{(1-\bar{F})\sigma_{a}^{2}}}\;,$$

where $cov({\hat{u}}_{w},{\hat{u}}_{p})$ is the covariance between estimated breeding values obtained using the whole dataset and the partial dataset, respectively. $\bar{F}$ is the average inbreeding coefficient in the validation population and $\sigma_{a}^{2}$ is the estimated genetic variance using each method. The average inbreeding coefficient of the validation set was computed using RENUMF90 of BLUPF90 software 2.53 [14] and was equal to 0.009.

Furthermore, we conducted a comparison of the models’ bias and dispersion using the LR method [15]. The bias is calculated as the difference between the average estimated breeding values (EBVs) of individuals in the validation set based on partial data and the whole data. The following formula was applied to determine the bias [17]:

(5) $${Bias}_{LR}=\bar{{\hat{u}}_{p}}-\bar{{\hat{u}}_{w}}$$

The dispersion was measured through the slope of the regression of ${\hat{u}}_{w}$ on ${\hat{u}}_{p}$ for the animals in the validation dataset [17]. The dispersion formula is given by

(6) $${Dispersion}_{LR}=\frac{cov({\hat{u}}_{w},{\hat{u}}_{p})}{var({\hat{u}}_{p})}$$

Additionally, the Spearman correlation between the estimated breeding values of the animals in the validation obtained by the three methods (LM, PLM, and PTM) was computed using R software [18]. Finally, the percentage of common animals in the top 20% was identified to evaluate potential changes in animals’ ranking.

## 3. Results and Discussion

### 3.1. Genetic Parameters

**Figure 1:**
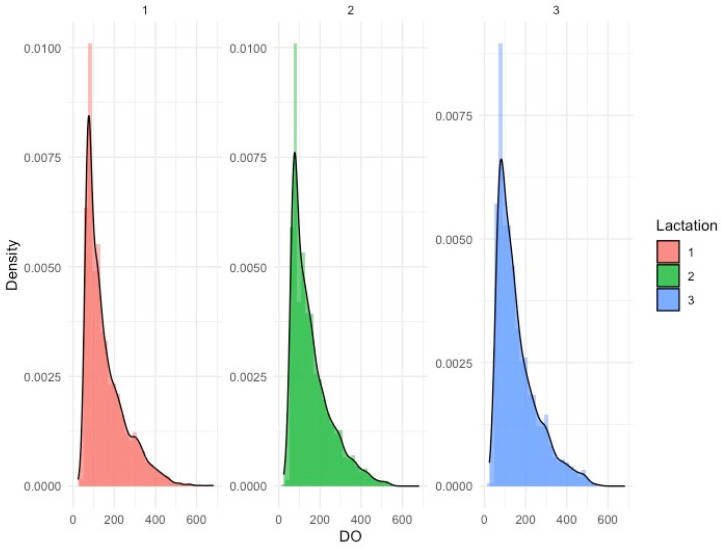
The distribution of days open (DO) across the three first parties.

A descriptive summary of the edited data with no censored records used in this study is presented in Table 1. The original dataset was reduced by 64% after data cleaning and processing were completed. The first parity contained more observations than other parities because of high culling rates due to fertility-related problems. The distribution of DO (Figure 1) is asymmetric and has a long tail to the right. The average DO was 155.46 ± 96.50 days for the first parity, 156.55 ± 94.10 days for the second parity, and, finally, 159.71 ± 96.81 days for the third parity. These numbers align closely with previous studies concerning Holstein cattle in the Mediterranean and arid areas [19,20]. The mean age at calving was 771.75 ± 77.81 days (25 months), 1201.27 ± 116.79 days (40 months), and 1628.29 ± 149.93 days (54 months) for the first, second, and third calving, respectively. Similar results were found for Holstein cows in similar climates [21,22]. Days in milk at first insemination ranged between 74.91 and 76.91 days across the first three lactations. This can be explained by the influence of the management of the farms. The third parity presents a somewhat larger standard deviation than the first two lactations. This might be explained by both a smaller number of observations and a possible larger rate of occurrence of clinical mastitis, levels of somatic cell count, and other health issues [23]. These health issues are shown to increase by parity and affect the variability of DO [24].

The heritabilities of DO (Table 2) ranged from 0.021 to 0.033 across the three different methods. These values are low but still in the range of what has been published in the literature [19,20,25]. The low heritability can be explained by the strong influence of the environment on the DO [26] on top of the shallow pedigree information [27]. PLM and PTM provided similar heritability results for DO, while LM with no censored records provided a slightly smaller value of 0.02. Garcia et al. [28] used six models to impute censored data and found out that the Gaussian linear model with penalty censored the Gaussian linear model, and the penalized threshold-linear model provided similar heritabilities for age at first calving and calving interval. Similar results were reported by Malhado et al. [29] and Costa et al. [13] for age at first calving in Nellore cattle. Despite similar estimates of heritability, the estimates of variance components have differed depending on the estimation method. The most noticeable difference was in the magnitude of all variance components between LM and both PLM and PTM. Posterior means of genetic, permanent environment, and residual variances for LM were the smallest. One potential explanation suggested by Hou et al. [12] is the overestimation of residual variances in models that account for censored records due to values beyond the upper limits. In both PLM and PTM, values were assigned equal to the maximum values of DO within each contemporary group plus 21 days. PTM provided higher additive genetic variance compared to that estimated with standard linear models, with or without a penalty, and a smaller residual variance compared to PLM. Similar results were found for Spanish Holstein cows by Gonzàlez-Recio et al. [30]. In fact, the authors reported that a censored Bayesian linear model is theoretically more appropriate than standard linear models for addressing censored data. However, it is important to construct contemporary groups carefully to avoid extreme-class problems (ECPs) where all observations in the same group are either censored or non-censored. Additionally, the genetic correlation between DO records and the binary trait indicating censorship status could influence the results, providing higher additive genetic variance in the PTM model.

### 3.2. The Prediction Accuracy of the Models

We assessed the prediction accuracy using the LR method [16] (Table 3). This approach can provide more accurate estimates of prediction accuracies than the predictive ability method as it does not require the adjusted phenotypes [31]. The overall accuracy rates obtained through the LR method were relatively low and no substantial differences were spotted among the methods. The predictive accuracy of LM (0.16) was somewhat smaller than that of PLM (0.21) and PTM (0.20). Different results were reported by Lázaro et al. [32] where LM exhibited a higher correlation between observed and predicted phenotypes (0.30), followed by PLM with an accuracy of 0.25 for age at first calving. Instead, Urioste et al. [11] reported that the threshold linear model had the highest Pearson correlations between sire breeding value predictions (0.67, 0.68, and 0.67 in the first, second, and third parity, respectively). The low accuracy found in this study can be attributed to the trait’s low heritability [12,33] and missing pedigree information in the studied population. According to Latifi and Nadiri [27], remarkably, these two factors influence the prediction accuracy. In fact, in simulated data of sex-limited traits with heritabilities 0.05, 0.1, and 0.2 and different rates of missingness of pedigree information, the lowest accuracy of prediction (0.018 ± 0.006) was for heritability 0.05 when 30% of the paternal pedigree was missing and 10% of the maternal pedigree was missing. Despite the low predictive accuracy (${\hat{ACC}}_{LR}$) of LM, the model yielded the lowest bias (−0.06) compared to PLM (−0.10) and PTM (−0.14) (Table 3). The negative bias of the latter two models suggests an underestimation of the true breeding values [34]. This finding was previously reported by Donoghue et al. [7] who claimed that the threshold linear model, when applied to high levels of censoring, slightly underestimates the records. The models’ slopes (${Dispersion}_{LR})$ were all found to be less than 1, with PTM having a marginally higher slope of 0.73. While a deviation from one may indicate a potential bias in the genetic evaluation, it could also be attributed to the small size of the validation cohort (2052 animals). Legarra and Revereter [16] noted that a dispersion lower than 1 is not always indicative of model quality; it may also depend on the number and relatedness of the animals in the validation set. Furthermore, the sources of biases related to statistical models in animal breeding may stem from various factors, such as the use of incorrect heritability, inaccurate modeling of the age effect, or improper definition of contemporary groups [34]. The results of our study showed that PLM and PTM methods produced similar prediction accuracy. However, when it came to variance components, using PTM resulted in a larger genetic variance and a smaller environmental variance, which led to a slightly higher estimated heritability. This difference may be due to the correlation between the censorship status variable and the DO trait. Additionally, both methods had similar bias and PTM had a slightly higher dispersion. Overall, PTM appears to be a promising method to address censored fertility data in this population. On the other hand, the LM approach had the lowest heritability, genetic variance, predictive accuracy, and dispersion. While the LM approach had a bias closer to 0, the low slope also suggests that there is a bias related to removing all censored data.

The Spearman correlation between the predicted breeding values of the validation dataset was 0.99 between PTM and PLM, 0.80 between PLM and LM, and 0.80 between PTM and LM. In the lower diagonal of Table 3, the ranking of animals varies at a higher magnitude between the penalty method, the penalized threshold model, and the Gaussian linear models. Similarly, Lázaro et al. [32] stated that the percentage of animals in common between the methods when selecting 10% of the individuals was 82.96% between LM and PM, 55.65% between LM and PTM, and 51.48% between PM and PTM, indicating that the largest changes in the ranking were spotted when using the threshold linear model. Different results were reported by Garcia et al. [28] where there were no significant changes in the ranking of the top 10% of Nellore bulls across the three methods.

## 4. Conclusions

Including fertility traits in conducting genetic evaluations is crucial for sustaining the profitability of dairy farms by selecting replacement heifers. Fertility field datasets often contain censored data for numerous reasons. In the context of this study where the dataset is limited, imputing censored records is important. Results for estimating genetic parameters for days open using LM, PLM, and PTM showed that the penalized threshold model marginally increased the trait’s heritability compared to linear models. However, the heritability estimates for all methods indicated a reduced genetic gain by selection. Both PLM and PTM yielded better prediction accuracy when using the LR method. Spearman correlations between the estimated breeding values of the validation dataset were high between PLM and PTM, explaining the large proportion of common animals in the top 20% of selected animals. The lack of changes in the ranking of animals between PLM and PTM suggests that both methods can be used to address censored data in this population.

## Figures and Tables

**Table 1:** Descriptive statistics of age at calving, days in milk at first insemination, and days open across the first three lactations: number of records, mean, standard deviation (SD), and minimum and maximum values in the uncensored dataset.

Parity	Number of Records	Age at Calving				Days in Milk at First Insemination				DO			
		Mean	SD	Min	Max	Mean	SD	Min	Max	Mean	SD	Min	Max
1	4183	771.75	77.81	553	993	75.89	27.46	22	499	155.46	96.50	37	683
2	2334	1201.27	116.79	880	1528	74.91	20.15	24	509	156.55	94.10	24	546
3	1033	1628.29	149.93	1196	2080	76.15	51.49	30	1556	159.71	96.81	30	550

**Table 2:** Posterior means and standard deviation for heritability and variance components for days open (DO) provided by different models. ($\sigma_{u}^{2}$ is the additive genetic variance; $\sigma_{pe}^{2}$ is the permanent environment variance; $\sigma_{e}^{2}$ is the residual variance; and $h^{2}$ is the heritability).

Method	$\sigma_{u}^{2}$	$\sigma_{pe}^{2}$	$\sigma_{e}^{2}$	$h^{2}$
LM	160.16 ± 77.04	363.87 ± 229.86	6766.4 ± 258.39	0.021 ± 0.010
PLM	389.22 ± 193.85	490.61 ± 376.20	12166.0 ± 389.59	0.029 ± 0.014
PTM	437.64 ± 217.97	705.76 ± 373.61	11932.0 ± 433.08	0.033 ± 0.016

**Table 3:** Estimates of ${Bias}_{LR}$, ${Dispersion}_{LR}$, and Spearman correlations between the estimated breeding values of the validation dataset using the three models LM, PLM, and PTM (above diagonal), and the percentage of animals in common in the top 20% of selected individuals (below diagonal). The background color mean that the cells are empty.

Method	LM	PLM	PTM
${\hat{ACC}}_{LR}$	0.16	0.21	0.20
${Bias}_{LR}$	−0.06	−0.10	−0.14
${Dispersion}_{LR}$	0.53	0.57	0.73
LM		0.80	0.80
PLM	52.67%		0.99
PTM	52.77%	96.34%	

## Data Availability

The datasets presented in this article are not readily available due to technical limitations. Requests to access the datasets should be directed to bouabidbadaoui@gmail.com.
